# A Rare Case of Functional Metastatic Follicular Thyroid Carcinoma With Concomitant Thyrotoxicosis

**DOI:** 10.1155/crie/1398125

**Published:** 2024-12-12

**Authors:** Yun Ann Chin, Zaheer Sumbul, Yi Lin Teh, Chiaw Ling Chng

**Affiliations:** ^1^Department of Endocrinology, Singapore General Hospital, Singapore, Singapore; ^2^Department of Nuclear Medicine and Molecular Imaging, Singapore General Hospital, Singapore, Singapore; ^3^Department of Medical Oncology, National Cancer Centre Singapore, Singapore, Singapore

**Keywords:** Graves' disease, metastatic follicular thyroid carcinoma, radioiodine treatment, thyroid stimulating hormone receptor antibody, thyroidectomy

## Abstract

We report a case of a 60-year-old lady with metastatic follicular thyroid carcinoma (FTC) who was presented with thyrotoxicosis and heart failure symptoms after total thyroidectomy. Clinical features and investigations led to the diagnosis of functional metastatic FTC with concomitant thyrotoxicosis. Levothyroxine therapy was stopped, and she was treated with propylthiouracil (PTU) followed by serial radioiodine treatments (RAITs) with good control of thyrotoxicosis and metastases. Despite having a very high disease burden with metastatic FTC, she has been able to maintain her functional status thus far, 4.5 years after initial diagnosis.

## 1. Introduction

Differentiated thyroid cancer (DTC) accounts for most thyroid cancers. Follicular thyroid carcinoma (FTC) constitutes 12% of DTC, second commonest after papillary thyroid carcinoma (PTC) [1]. Distant metastasis accounts for 7%–23% of all patients with FTC [2]. Functional metastatic FTC is even rarer. However, the overall survival rates in metastatic disease with thyrotoxicosis are similar to those of patients with metastatic disease who are euthyroid [2]. Very few cases of concomitant functional FTC and Graves' disease (GD) are reported in the literature. The presence of GD exerts an adverse impact on FTC, contrary to previous beliefs [3]. In this case, we highlight the importance of maintaining a high index of suspicion for the possibility of functional metastases causing thyrotoxicosis in patients with extensive metastatic FTC after total thyroidectomy. In addition, the management strategies of this challenging case are discussed in detail in this report.

## 2. Case Presentation

**Figure 1:**
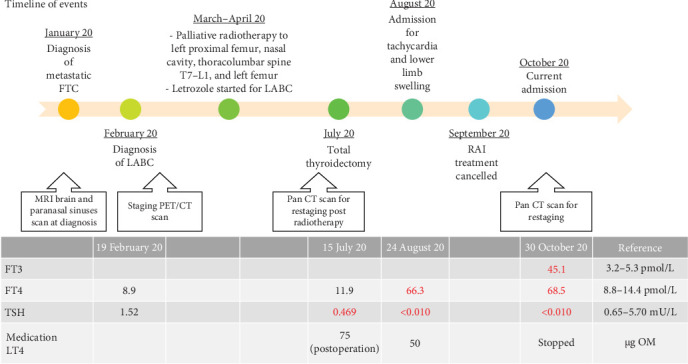
Timeline of events with thyroid function test trend. CT, computed tomography; FT3, serum free tri-iodothyronine; FT4, serum free thyroxine; FTC, follicular thyroid carcinoma; LABC, locally advanced breast cancer; LT4, levothyroxine; MRI, magnetic resonance imaging; OM, every morning; PET, positron emission tomography; RAI, radioactive iodine; TSH, serum thyroid stimulating hormone.

A 60-year-old Chinese lady was diagnosed with metastatic FTC when she presented in January 2020 with 3 months history of bilateral hearing loss (Figure 1). Magnetic resonance imaging (MRI) brain and paranasal sinuses showed a large enhancing heterogeneous mass with lobulated margins involving bilateral sphenoid regions with extension into the nasal cavities, as well as the anterior and middle cranial fossa. Incidentally, large left thyroid nodules, with the largest nodule measuring 3.6 cm × 2.6 cm × 2.5 cm, with enhancing left level III nodes were seen on the MRI scan. Histology of the intranasal tumor obtained via examination under anesthesia and biopsy showed metastatic thyroid follicular carcinoma, with Ki67 proliferation index 5%–7%. Ultrasound guided fine needle aspiration (FNA) of left thyroid nodules and lymph nodes showed atypia of undetermined significance/follicular lesions of undetermined significance (AUS/FLUS; Bethesda III) and presence of follicular cells respectively. She was diagnosed with follicular carcinoma from the thyroid with metastasis to the lymph nodes, nasal cavity, and brain.

Staging positron emission tomography/computed tomography (PET/CT) scan was done and showed multiple hypermetabolic lytic lesions in the axial and appendicular skeleton with at least moderate–severe spinal canal stenosis and pathological left femoral neck fracture. She underwent nailing of the left proximal femur with bone biopsy for the pathological left femoral neck fracture (2 months after diagnosis). Histology of the left proximal femur was in keeping with metastatic FTC.

She was advised to undergo total thyroidectomy, but she initially declined. She opted for palliative radiotherapy to the left proximal femur, nasal cavity, thoracolumbar spine T7-L1, and left femur from March to April 2020 after the nailing of the left proximal femur.

**Figure 2:**
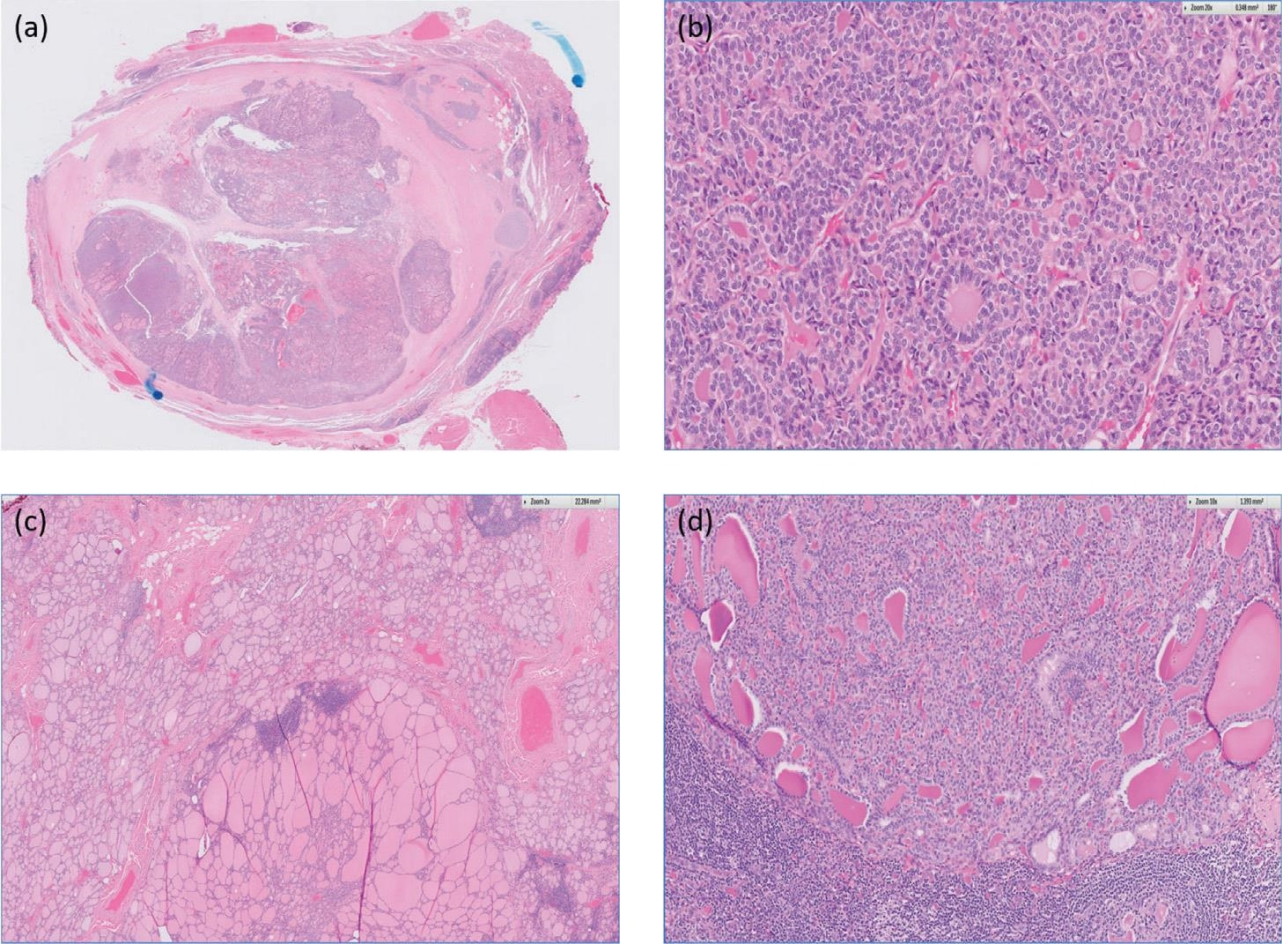
Histology using H&E staining. Thyroid. (a) Section of the tumor showed a thick fibrous capsule with multiple foci of capsular invasion. (b) High power ×20 resolution showed tumor cells arranged predominantly in microfollicles. (c) The background thyroid tissue showed lymphocytic thyroiditis with lymphoid follicles (resolution ×2). An adenomatoid nodule was present. (d) Lymph node metastasis. Section under ×10 resolution showed metastatic tumor in the subcapsular sinus of a lymph node. H&E, hematoxylin and eosin.

She finally agreed and underwent total thyroidectomy and left selective neck dissection (II–IV) in July 2020. Histology from thyroid specimen showed pT3aN1b follicular carcinoma, widely invasive, and 3 out of 26 lymph nodes were positive for malignancy (Figure 2). No features of poorly differentiated histology. Her thyroid function showed subclinical hyperthyroidism a day prior to the total thyroidectomy (Figure 1). The primary physician attributed that to nonthyroidal illness as she was clinically euthyroid and was very ill at that time. She was started on levothyroxine (LT4) replacement (1.7 μg/kg/day) postoperatively. She was planned for radioiodine treatments (RAITs) with steroids cover under thyroxine withdrawal in 3 months postthyroidectomy.

She was readmitted 3 months postthyroidectomy (prior to scheduled RAIT) for palpitations and shortness of breath (Figure 1). It was associated with bilateral lower limb swelling and reduced effort tolerance. She also complained of heat intolerance, loss of weight of 10 kg, and diarrhea over 3 months.

**Figure 3:**
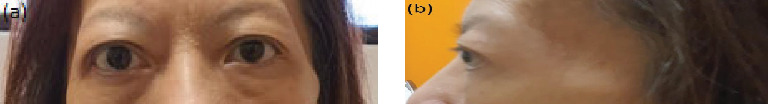
The patient's eyes showing bilateral lid retraction. (a) Front view. (b) Side view showing proptosis. (Patient consented to the publication of photo).

On examination, she was tachycardic (146 bpm) and tachypnea (28 breaths per min). The rest of the vital signs were unremarkable. She had bilateral mild proptosis and lid retraction, but no chemosis or diplopia (Figure 3).

She had fine tremors and sweaty palms. There was a well-healed thyroidectomy scar. There were decreased breath sounds and stony dullness to percussion over the right base of the lung and bilateral pitting edema up to the mid-shin.

Investigations showed biochemical primary hyperthyroidism (serum thyroid stimulating hormone (TSH) <0.010 mU/L and serum free thyroxine (FT4) 68.5 pmol/L (normal range [NR] 8.8–14.4 pmol/L)). Her liver panel showed cholestasis without hyperbilirubinemia, which could be related to thyrotoxicosis or high bone turnover. She had parathyroid hormone (PTH)-independent hypercalcemia, which could be due to malignancy, bone metastasis, or thyrotoxicosis. She also had normochromic normocytic anemia, which was at her baseline 8–10 g/dL, likely secondary to anemia of chronic disease from thyrotoxicosis, malignancy, and bone marrow suppression from bone metastasis. Other biochemistry tests were unremarkable (Table 1).

Other investigations showed features of pulmonary edema; raised serum pro-B-type natriuretic peptide (BNP) and increased perihilar haziness on chest radiography as well atrial flutter on electrocardiography (ECG). Echocardiography showed a moderately enlarged left atrium with preserved ejection fraction of 79% with no thrombus. A repeat whole-body (pan)-CT scan showed persistent and stable disease burden.

She was referred to the Endocrinology Unit at this point. A TSH receptor antibody (TRAb) level was performed in view of the presence of signs of thyroid eye disease (TED), which was high at 26.10 IU/L (NR <1.76 IU/L) confirming concomitant GD. TRAb was not measured prior to thyroidectomy for comparison. She was diagnosed with functional metastatic FTC on the background of GD, complicated by atrial flutter and congestive heart failure. The thyrotoxicosis could either be from relapse of GD postsurgery or functional metastatic FTC or a combination of both.

**Figure 4:**
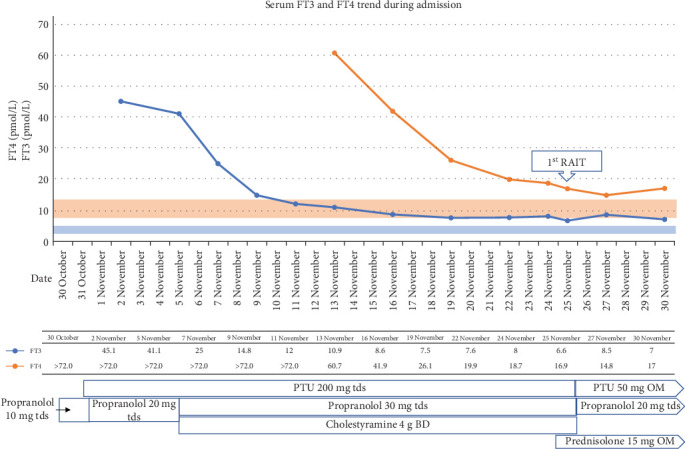
Serum FT3 and FT4 trend during admission. Orange box: normal range for serum FT4 and blue box: normal range for serum FT3. BD, twice a day; FT3, serum free tri-iodothyronine; FT4, serum free thyroxine; OM, every morning; PTU, propylthiouracil; RAIT, radioiodine treatment; tds, three times a day.

She was not deemed to be in thyroid storm based on clinical assessment at presentation. The LT4 was promptly stopped and she was initiated on high dose oral propylthiouracil (PTU), cholestyramine, and propranolol (Figure 4).

Although steroid may reduce the thyroid hormone (TH), in particular tri-iodothyronine levels further, we did not prescribe as she was frail and was at risk of infection. We aimed to institute RAIT after optimizing her TH and clinical condition.

For the treatment of atrial flutter, she underwent a cardioversion which reverted her back to sinus rhythm. Thereafter, she was started on warfarin, titrated to prothrombin time-international normalized ratio (PT-INR) target of 2.0–3.0. She was also treated with intravenous frusemide and started on fluid restriction for the congestive heart failure.

**Figure 5:**
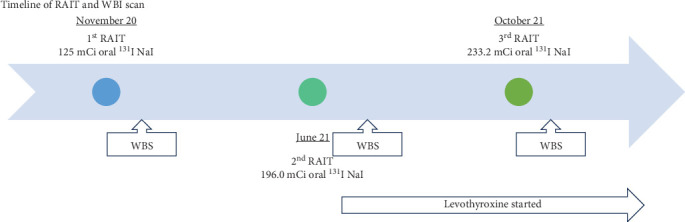
Timeline of RAIT and WBS. NaI, sodium iodide; RAIT, radioiodine treatment; WBS, whole body scan.

She received 125 millicurie (mCi) oral ^131^I sodium iodide (NaI) solution for her first RAIT 1 month after admission under steroid prophylaxis (Figures 4 and 5) as a preemptive to avoid worsening of TED and to prevent edema of skull base metastases. The PTU and propranolol doses were reduced subsequently.

**Figure 6:**
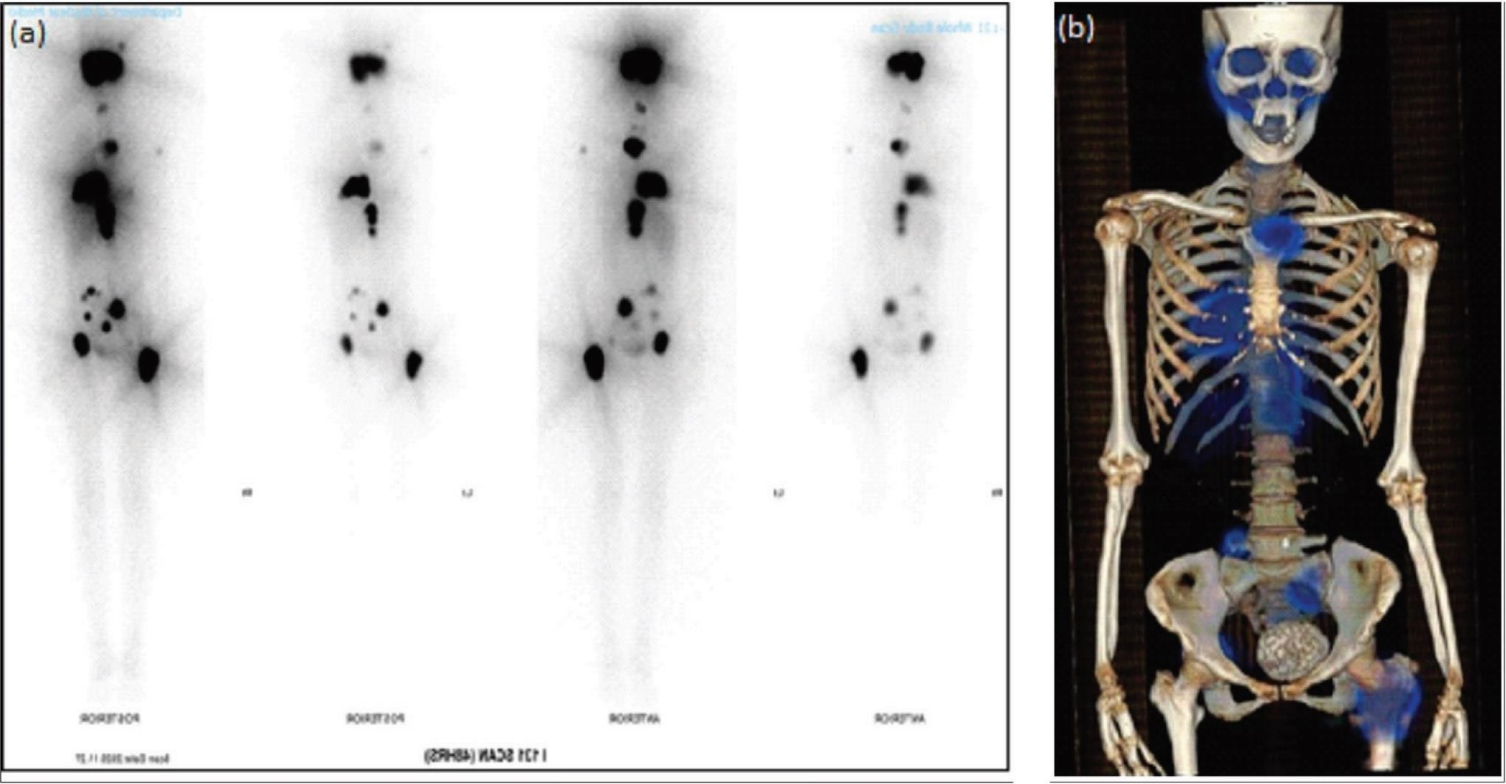
(a) Whole body iodine scan showed multiple intensely ^131^I avid foci scattered at the skull, chest, abdomen, pelvis, and proximal upper and lower extremities. (b) 3D image reconstruction of SPECT/CT scan. SPECT/CT scan showed destructive osseous lesions in the base of skull extending into the middle cranial fossa, nasopharynx, bilateral sinonasal cavities, masticator spaces and orbits, manubrium, left proximal humerus, right 9th rib, thoracolumbar spine with spinal canal involvement, sacrum, left hemipelvis, and bilateral proximal femora. SPECT, single-photon emission computerized tomography.

Post-RAIT, whole body scan (WBS) showed multiple intensely ^131^I avid foci scattered at the skull, chest, abdomen, pelvis, and proximal upper and lower extremities on the WBS (Figure 6a), consistent with sites of metastatic disease on the single-photon emission computerized tomography (SPECT)/CT scan (Figure 6b).

**Figure 7:**
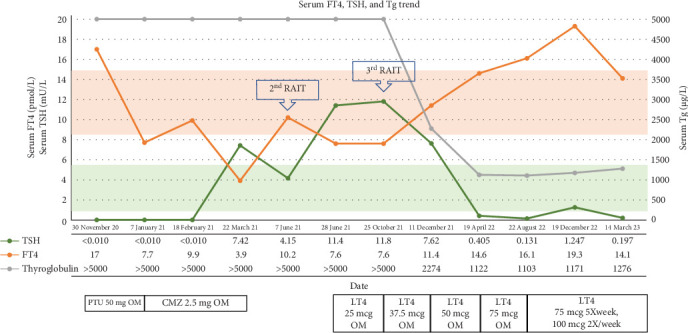
Serum FT4, TSH, and Tg trend. Orange box: normal range for serum FT4 and green box: normal range for serum TSH and TgAb <0.9 IU/ml (NR <4.0). CMZ, carbimazole; FT4, serum free thyroxine; LT4, levothyroxine; NR, normal range; PTU, propylthiouracil; TSH, serum thyroid stimulating hormone.

The antithyroidal medication was tapered and discontinued 4 months after the first RAIT. She underwent further outpatient second RAIT with 196.0 mCi oral ^131^I NaI solution, and third RAIT with 233.2 mCi oral ^131^I NaI solution 6 months and 11 months after the first RAIT (Figures 5 and 7). Her latest post RAIT WBS done 3 days post third RAIT showed less intense tracer uptake at the anterior neck and no new ^131^I avid lesions, although there were still multiple ^131^I avid metastases in the axial and appendicular skeleton.

Levothyroxine was started after her second RAIT, aiming to keep serum TSH <0.1 mU/L (Figure 7). The thyroglobulin level fell from >5000 μg/L to approximately 1200 μg/L post third RAIT (Figure 7). Her TRAb level also improved progressively and was undetectable 6 months after the first RAIT.

She is still under regular follow-up by a multidisciplinary team of endocrinologist, nuclear medicine physician, and medical oncologist and remains activities of daily living (ADL) independent.

## 3. Discussion

DTC causing thyrotoxicosis is very rare, with only about 70 cases reported in the literature [4]. Majority of the functional thyroid carcinoma is associated with FTC. Functional thyroid carcinoma can be classified into primary hyperfunctioning thyroid carcinomas, occurring in the thyroid primary itself or metastatic hyperfunctioning thyroid carcinoma, usually due to widespread large tumor metastases [5]. FTC constitutes 46.5% of primary hyperfunctioning thyroid carcinoma and 71.4% of metastatic hyperfunctioning thyroid carcinoma [5]. It is often difficult to differentiate hyperfunctioning thyroid carcinoma from benign autonomous functional thyroid nodules, as they share similar features; clinical thyrotoxicosis with hot nodules on thyroid scintigraphy. The suspicion of primary hyperfunctioning thyroid carcinoma is based on several characteristics: no improvement in thyrotoxicosis following RAIT and features of malignancy in the thyroid nodule, such as development of hypoechoic solid nodules with microcalcifications on ultrasound examination, increase in tumor size over a short time period, fixation of the tumor to adjacent structures, signs/symptoms of tumor invasion, and risk factors for thyroid malignancy such as history of head and neck irradiation [5]. On the other hand, metastatic hyperfunctioning thyroid carcinoma is suspected in thyrotoxic patients with a high number of metastatic lesions from pan imaging or a history of total thyroidectomy [5, 6]. In this case, the patient had metastatic hyperfunctioning thyroid carcinoma. Majority (60%) of such thyroid carcinoma are diagnosed with thyrotoxicosis concomitantly, while the rest have thyrotoxicosis occurring between 1 month and 15 years from the diagnosis of thyroid carcinoma [6]. The exact pathogenesis of thyrotoxicosis by metastatic FTC remained unknown. Postulated mechanisms include: (1) a large aggregate tumor mass functioning autonomously and (2) somatic mutations in TSH receptor genes leading to activation of the intracellular cAMP cascade leading to hyperthyroidism [4, 7].

The incidence of concomitant thyroid cancer and GD ranges between 1.7% and 2.5% [8]. GD has been diagnosed biochemically post thyroidectomy, with a variable, latent time to manifestation (3–120 months) [9].

In this case, the patient developed thyrotoxicosis, with elevated TRAb level after thyroidectomy. Aside from the elevated TRAb level, the patient had mild signs of TED and histology showed lymphocytic thyroiditis. The patient could have euthyroid GD before thyroidectomy. Thyroidectomy might have resulted in the positive TRAb. Several postulations on the mechanisms include surgery destroys thyroid cells causing increase in TSH receptor expression, surgery induces an immune system response, such as the stimulation of the antigen-presenting cells that control the activation of suppressor or regulatory cells, stress from general anesthesia, and surgery causes neuroendocrine fluctuations that disrupt immunological homeostasis as well as postoperative bacterial and viral infections increase the number of cluster of differentiation (CD)5+ B cells, which stimulate the TSH receptor antibodies leading to GD [10].

Aside from being a diagnostic marker of GD, elevated TRAb level is associated with clinically more aggressive thyroid carcinoma, higher rates of multifocality and local invasion, and increased rates of metastases [4]. GD with a background of multinodular goiter carries an increased risk of malignancy compared to diffuse goiter [4]. Both the TSH and TRAb activate adenylate cyclase and phospholipase C cascade, with mitogenic and antiapoptotic effects causing normal thyroid tissue to become hyperplastic and hyperfunctioning leading to tumor genesis as well as growth and metastatic spread of thyroid carcinoma [8]. Suzuki, Nakagawa, and Aizawa [11] postulated that the remnant normal thyroid tissue postthyroidectomy in individuals with GD may trigger an autoimmune response sensitizing the antibodies that would stimulate cancer cells leading to thyrotoxicosis. Besides autonomous TH production by the carcinoma itself, TSH receptor on the metastatic tumor can function as an antigen of TRAb, stimulating the production of TH [8]. Hence, in this case, the mechanisms of thyrotoxicosis are likely from the stimulation of TH by elevated TRAb and the large tumor mass functioning autonomously. The elevated TRAb level in GD perpetuates more severe thyrotoxicosis in patients with functional metastatic FTC.

The primary aim in the management of functional thyroid carcinoma is to control thyrotoxicosis and eradicate the carcinoma itself. Therefore, total thyroidectomy is the first line management for metastatic thyroid carcinoma as it eliminates the primary thyroid carcinoma and reduces the metastatic lesions require ^131^I treatment [5]. Debulking surgery of large and functioning metastatic lesions can also be considered, especially if the lesions are resistant to RAIT and are resectable. This is essential to allow concentration of radioactive iodine (RAI) to the remaining RAI avid lesions.

RAIT with antithyroid medications can treat thyrotoxicosis and decrease the size of metastatic lesions with remission rates of about 33% [8, 12]. High doses of antithyroid medication are often needed to control the hyperthyroidism prior to RAIT to prevent thyroid storm, which can be life-threatening [12, 13]. However, it may be impossible to aim for TSH>30 mU/L required under usual circumstances in radioiodine ablation of thyroid cancers in view of both functional metastases with concomitant GD. Nonetheless, it was noted by Haq et al. [13] that hyperfunctioning metastases can sequester large quantities of ^131^I even in the presence of suppressed TSH levels. In addition, functioning metastases retain ^131^I for a longer period than those with nonfunctioning metastases. Consequently, those who respond well to RAIT are associated with better outcomes [12]. Antithyroid medications not only reduce TH via inhibition of the effects of thyroid peroxidase, but they also exert an immunosuppressive effect via reduction in the number of circulating activated T helper/inducer cells and increment in the number of circulating activated T suppressor/cytotoxic, leading to inhibition of the initiation and progression of thyroid carcinoma [8]. Antithyroid medications and RAIT also improve TRAb level due to the destruction of tumor cells containing TSH receptors, which are antigen for TRAb.

About 40% of the mortality occurs within a month of onset of thyrotoxicosis and shortly after operation or RAIT in functional metastatic FTC [12]. This is partly due to the massive release of preformed TH from destructive thyroiditis after RAIT. Hence, a short course of steroids initiated during this period may help mitigate the steep rise in TH post-RAIT. The overall survival rates in metastatic disease with thyrotoxicosis are similar to patients with metastatic disease who were euthyroid [14, 15].

## 4. Conclusion

We described an unusual case of a patient who presented with severe thyrotoxicosis after total thyroidectomy for metastatic FTC with presence of TRAb. Functional metastatic FTC is associated with poor prognosis. High index of suspicion and prompt identification of thyrotoxicosis is crucial to ensure targeted treatment for both the thyrotoxicosis and metastases.

## Figures and Tables

**Table 1:** Initial laboratory investigations on admission.

Laboratory tests	Values	Reference range
Serum urea	3.9	2.7–6.9 mmol/L
Serum sodium	140	136–146 mmol/L
Serum potassium	4.3	3.6–5.0 mmol/L
Serum chloride	106	100–107 mmol/L
Serum bicarbonate	26.2	19.0–29.0 mmol/L
Serum glucose	5.2	3.9–11.0 mmol/L
Serum creatinine	21	37–75 μmol/L
Serum total protein	56	68–85 g/L
Serum albumin	**27**	40–51 g/L
Serum total bilirubin	15	7–32 μmol/L
Serum alkaline phosphatase	**249**	39–99 U/L
Serum alanine transaminase	28	6–66 U/L
Serum aspartate transaminase	**46**	12–42 U/L
Serum gamma glutamyl transferase	**171**	9–53 U/L
Serum magnesium	0.83	0.74–0.97 mmol/L
Serum calcium	2.27	2.09–2.46 mmol/L
Serum calcium (corrected)	**2.55**	2.09–2.46 mmol/L
Serum phosphate	1.31	0.94–1.50 mmol/L
Serum intact PTH	3.3	0.9–6.2 pmol/L
Serum troponin T	16	<30 ng/L
Serum NT-proBNP	**3761**	<150 pg/ml
TSH	**<0.010**	0.65–3.70 mU/L
Serum free thyroxine	**68.5**	8.8–14.4 pmol/L
Hemoglobin	**10.8**	11.5–15.0 g/dL
WBC count	5.98	4.0–10.0 × 10^3^ μL
Platelet count	268	150–450 × 10^3^ μL

*Note*: The bolded values signify out of normal range levels.

Abbreviations: BNP, B-type natriuretic peptide; PTH, parathyroid hormone; TSH, serum thyroid stimulating hormone; WBC, white blood cell.

## Data Availability

Research data are not shared.
